# Characteristics of humoral and cellular responses to coronavirus disease 2019 (COVID-19) inactivated vaccine in central China: A prospective, multicenter, longitudinal study

**DOI:** 10.3389/fimmu.2023.1107866

**Published:** 2023-03-03

**Authors:** Youhua Yuan, Junhong Xu, Bing Ma, Guohua Chen, Zhibin Wang, Shanmei Wang, Nan Jing, Jiangfeng Zhang, Baoya Wang, Wenjuan Yan, Qi Zhang, Qiongrui Zhao, Yi Li

**Affiliations:** ^1^ Department of Clinical Microbiology, Henan Provincial People’s Hospital, People’s Hospital of Zhengzhou University, and People’s Hospital of Henan University, Zhengzhou, Henan, China; ^2^ Department of Laboratory, Zhengzhou Municipal Chinese Medicine Hospital, Zhengzhou, Henan, China; ^3^ Department of Geotechnical Engineering, Henan Electric Power Survey and Design Institute, Zhengzhou, Henan, China; ^4^ Center of Clinical Research Service, Henan Provincial People’s Hospital, People’s Hospital of Zhengzhou University, and People’s Hospital of Henan University, Zhengzhou, Henan, China

**Keywords:** cellular immune response, COVID-19, inactive vaccine, kinetics, neutralizing antibody

## Abstract

**Introduction:**

In China, the long-term immunogenicity and adverse effects of inactivated vaccines produced by different or the same manufacturer remain unclear. Therefore, the objective of this study was to evaluate the cellular immune responses and neutralizing antibody kinetics of homologous and heterologous administrations of an inactivated coronavirus disease 2019 (COVID-19) vaccine 240 days after the second vaccination.

**Methods:**

This prospective, multicenter, observational, longitudinal study involved 595 participants with a negative SARS-CoV-2 polymerase chain reaction result who were serologically tested and followed for 8 months after vaccination. Neutralizing antibodies, interferon-gamma (IFN-γ), interleukin (IL)-6, CD4+ T-lymphocyte, and B-lymphocyte counts were evaluated in serum samples after stimulation with 2 μg/mL SARS-CoV-2 spike protein for 16 h at follow-up intervals of 2 months.

**Results:**

Most participants [582/595; 146 male participants, 449 female participants; mean age 35 (26–50 years)] rapidly developed neutralizing antibodies after two doses of the vaccine administered 3-weeks apart. The positive rate of neutralizing antibodies peaked at 97.7% at 60–90 days, decreased, and stabilized at 82.9% at 181–240 days post-vaccination. Lower antibody concentrations were correlated with older age, longer duration after vaccination, non-health care workers, mixed-manufacturer vaccinations, and intervals of less than 40 days between two doses of vaccination, whereas lower IFN-γ levels and B-lymphocyte counts were associated with older age, blood type A, and non-health care workers. A higher IL-6 level was associated with older age, mixed-manufacturer vaccinations, intervals of less than 40 days between two doses of vaccination, and medical staff. Adverse reactions were mild or moderate and self-limited, with no serious events reported.

**Discussion:**

Two doses of the Chinese inactivated vaccine induced robust and rapid antibody expression and cellular immune responses. Boosting vaccination is considered important, as antibodies and cellular immune responses were reduced in susceptible populations.

## 1 Introduction

To date, coronavirus disease 2019 (COVID-19), a newly emerging infectious disease caused by severe acute respiratory syndrome coronavirus 2 (SARS-CoV-2), has infected over 600 million people worldwide and killed over 6 million people (1, 2). To control the COVID-19 epidemic and build an active immunization barrier among populations, the Chinese Government provided free COVID-19 vaccination to all citizens as of December 2020 (3). The population required to be vaccinated included children aged 3–11 years, adolescents aged 12–17 years, and adults. Booster shots were administered to people at least 6 months after their previous vaccination on October 20, 2021 (4–9). The full coverage rate of the COVID-19 vaccination program has reached approximately 90% of the population required to be vaccinated in 2022 (10).

Four types of COVID-19 vaccine have been approved for use worldwide: mRNA, inactivated virus, adenovirus, and recombinant protein vaccines (11). In China, the latter three have been approved and are produced by eight manufacturers. Among them, Beijing Kexing Zhongwei, Beijing Kexing, Beijing Biology, Lanzhou Biology, Wuhan Biology, and Changchun Biology produce the inactivated vaccine type for a vaccination program of two doses at an interval of 3–8 weeks. Tianjin CanSino produces the adenovirus vaccine type for a vaccination program of one dose and Anhui Zhifei produces the recombinant protein vaccine type for a vaccination program of three doses at intervals of 1 month (12). To date, most of the Chinese population has been vaccinated with the inactivated vaccine. Vaccination is the most effective measure to reduce mortality from COVID-19 and serious diseases (12); however, vaccines are scarce in certain countries and some regions in China (13). Therefore, the mixing of vaccine brands between doses was considered a feasible strategy to complete the entire course of basic immunization with two doses of the inactivated COVID-19 vaccine. Research in other countries has shown that the antibody titer of individuals vaccinated with a different type of vaccine 28 days after the first vaccination was higher than that of individuals vaccinated with a vaccine from the same manufacturer for both doses, without an increase in adverse reactions (14). However, in China, data on long-term immunogenicity and adverse effects of inactivated vaccines produced by different manufacturers or the same manufacturer have not been reported.

Therefore, we designed a multicenter, longitudinal, observational study to evaluate the immunogenicity and reactogenicity of a second dose of inactivated vaccine among those who received the two vaccines from different manufacturers. Our findings could provide evidence for an appropriate vaccination strategy against COVID-19.

## 2 Materials and methods

### 2.1 Ethics statement

The study complied with the principles of the Declaration of Helsinki and Good Clinical Practice, and was approved by the Ethics Committee of Henan Provincial People’s Hospital (approval number 20210051, date of approval May 24, 2021).

### 2.2 Study design and participants

This study was a multicenter, longitudinal, prospective, observational study conducted in two hospitals (Henan Provincial People’s Hospital and Zhengzhou Municipal Chinese Medicine Hospital) and one institution (Henan Electric Power Survey and Design Institute) in central China.

Between June 19, 2021 and April 30, 2022, healthy or clinically stable adults (aged 18–80 years) who had received two doses of the inactivated COVID-19 vaccine between 3 and 8 weeks (21–56 days) before the screening visit were recruited. Participants with documented reverse transcription-polymerase chain reaction-confirmed COVID-19 or those who had been vaccinated with any other vaccine (e.g., influenza or others) or booster vaccine since the primary dose were excluded. Participants with a clinically notable acute illness or a body temperature of at least 38°C within 24 h before receiving the planned dose of the study vaccine, clinical manifestations compatible with those of COVID-19, and any condition that contraindicates or discourages the administration of an inactivated vaccine, including pregnancy, were excluded (15).

The full eligibility criteria were provided in the volunteer recruitment notification distributed through social networks, including WeChat groups. Interested candidates contacted one of the study institutions directly; at this point, a personal interview was scheduled to explain the study and verify the selection criteria. All participants provided their informed consent in writing before enrollment.

### 2.3 Experimental procedures

The inactivated vaccine was administered as a single intramuscular injection for each of the two approved doses. All participants were clinically evaluated and blood samples were collected for immunological evaluation on day 0. Follow-up visits were scheduled on days 1–30 to measure vital signs, review any solicited and unsolicited adverse events, and update medical and medication records. At intervals of 2 months, 8 mL of blood samples was collected from volunteers and distributed into four tubes (2 mL/tube) with or without the EDTA anticoagulant (two tubes for each) to detect neutralizing antibodies and cellular immune response parameters. The two tubes with the EDTA anticoagulant were used to determine lymphocyte subsets with or without antigen-stimulated SARS-CoV-2 spike peptides, and the two tubes without the EDTA anticoagulant were used to determine neutralizing antibodies against SARS-CoV-2 and cytokines with or without antigen-stimulated SARS-CoV-2 spike peptides. The duration of the immune response was determined for each participant as the time interval between the date of blood collection and that of the second dose of the vaccine. As the study is ongoing, further follow-up data on the third dose of a vaccine booster will be reported in the future. The participants in the study (mixed-manufacturer vaccination for the two doses) and control (same-manufacturer vaccination for both doses) groups remained in local community health centers for at least 30 min after vaccination to monitor safety, and the occurrence of any adverse event during this observation period was recorded. In cases of severe adverse events, the investigator contacted the participant, and the intensity of adverse events was graded according to the adverse events severity scale as follows: 1, mild; 2, moderate; 3, severe; or 4, life-threatening. Safety definitions and a list of solicited adverse events were defined as previously described (16, 17).

Antigen-specific humoral immune responses were detected using a commercial enzyme-linked immunosorbent assay (ELISA) anti-SARS-CoV-2 S kit (Shanghai GeneoDx Biotech, Ltd., Co., Shanghai, China), which detects neutralizing immunoglobulin G (IgG) antibodies against the SARS-CoV-2 spike protein receptor-binding domain (RBD) on a universal microplate reader (DNM-9602; Beijing Pulang Ltd., Co., Beijing, China); values > 6.5 IU/mL were considered positive. According to the manufacturer’s instructions, values > 100 IU/mL were considered as 100 IU/mL. Parameters of cell immune responses were evaluated by quantifying the levels of interferon (IFN)-γ, interleukin (IL)-6, and CD4, and counts of B-lymphocyte subsets in the plasma after overnight stimulation of whole blood with 2 μg/mL SARS-CoV-2 spike peptides (Wuhan Huamei Biotechnology Ltd., Co., Wuhan, China) or dimethyl sulfoxide in whole-blood culture as a control, requiring only 2 mL blood (14). Cytokine levels and lymphocyte subset counts were measured using flow cytometry (Beamcyte-1026; Changzhou Beamdiag Co., Ltd., Changzhou, China). ABO blood typing was performed using a test-tube method according to the manufacturer’s protocol (Chengdu Xiehe Ltd., Co., Chengdu, China).

### 2.4 Statistical analysis

Factors that influence the concentration of neutralizing antibodies and cellular responses were analyzed using a multivariate linear regression model, which could be set on one of the classified variances as a reference to calculate the *B* value (18). Because these detected records included missing data for neutralizing antibodies and cellular immune responses, we used mixed linear models that can handle unequal numbers of repeated observations for individuals when there were random missing data. To analyze the changes in neutralizing antibodies and cellular immune responses over time, we used a mixed linear model with continuous natural log_10_-transformed cellular immune response data or the log_2_-transformed concentration of neutralizing antibodies against SARS-CoV-2 spike protein between days 1 and 240 as the dependent variable; age, sex, body mass index (BMI), occupation, vaccination mode, duration since the second dose, interval between doses of vaccination, and ABO blood were included as covariates in the models.

Based on their age, we divided the participants into three groups, namely, 18–30, 31–50, and >50 years old. Furthermore, based on the study of Lusting et al. (19, 20), we divided participants into three BMI groups: <18.5, 18.5–23.9, and >23.9 kg/cm^2^. Based on the Chinese COVID-19 inactivated vaccine shot procedure (3–8 weeks), we divided the interval between two doses of the vaccination for the participants into two groups: 21–40 and >40 days. Based on the duration after vaccination of the participants, we divided the duration after vaccination of the participants into eight groups: 1–14, 15–30, 30–60, 61–90, 91–120, 121–150,151–180, and 181–240 days. In addition, other variables, including sex, occupation, vaccination type, and blood type, based on their natural classification, were divided into different categories. The mixed model distribution curves of log_2_-transformed neutralizing antibodies and log_10_-transformed cellular response with duration adjusted by age, sex, BMI, occupation, vaccination mode, duration since the second dose, interval between doses of vaccination, and ABO blood type were plotted using Prism 8.0 (GraphPad Software Inc., USA). Although the statistical analysis controlled for potential confounders, we included variables that showed significant associations with humoral and cellular responses in the mixed model.

Reactogenicity analysis results are presented as percentage of participants. Both primary and safety analyses included all participants who received two doses of the inactivated vaccine and had suffered local and systemic adverse events for 30 consecutive days after vaccination. An independent data-monitoring committee, comprising independent scientists not involved in this study, has been reviewing the data regularly for safety and scientific integrity. All analyses, including linear regression, linear mixed models, and adverse effect analysis, were conducted using SPSS 25.0 (IBM Corporation, Armonk, NY, USA) and plotted using Prism 8.0.

## 3 Results

### 3.1 Basic characteristics of study participants

**Figure 1:**
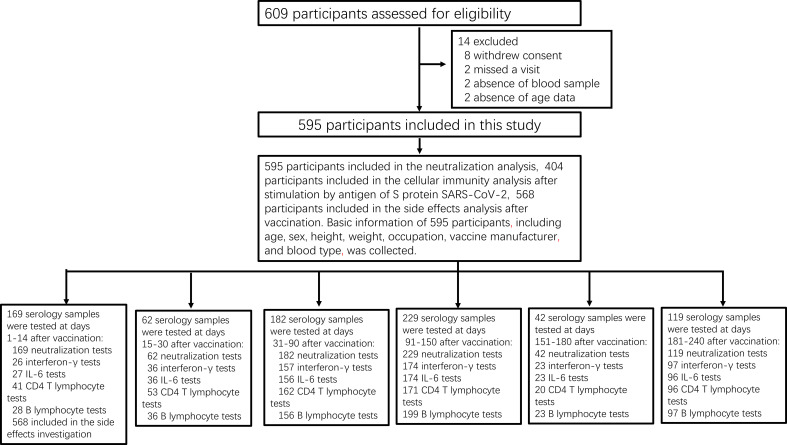
Study profile. Prospective cohort of Chinese individuals immunized with the inactivated vaccine and serological assays. Following vaccination, the participating medical staff of Henan Provincial People’s Hospital and Zhengzhou Municipal Traditional Medicine Hospital, and workers of the Henan Electric Power Exploration Company in central China were followed up monthly for 8 months between June 19, 2021, and April 30, 2022.

A total of 1,350 serum samples were collected from 595 participants (**Figure 1**), of whom 457 (76.8%) were medical staff and 138 (23.2%) were workers of the Henan Electric Power Exploration Company in central China. We excluded 14 (2.4%) participants from the regression analysis and mixed model analysis due to missing data: 8 who withdrew their consent, 2 who did not answer age-related questions, 2 who missed a follow-up visit, and 2 who did not provide blood samples (**Figure 1**). The concentration kinetics of IgG neutralizing antibodies were evaluated against the RBD for all study participants at least once during the 8-month timeframe and at least four times for 119 participants (20%). We also evaluated CD4+ T-lymphocyte counts, B-cell counts, and IFN-γ and IL-6 levels after stimulation with the S protein of SARS-CoV-2 in 543, 539, 513, and 512 individuals, respectively. Except for blood type, all demographic and baseline characteristics were balanced between the intervention and control groups (**Table 1**). The median age at vaccination was 35 years (interquartile range [IQR]: 28–50) for those administered a vaccine of the same manufacturer (control) and 33 years (IQR: 21–50) for those administered vaccines from different manufacturers (intervention) (**Table 1**).

**Table 1:** Baseline characteristics of the study participants (N = 595).

Factor	Mixed manufacturer (n = 435)	Same manufacturer (n = 160)	*p*	Overall
Sex				
Men	110	36	0.507	146
Women	325	124		449
Age (years), M (P25, P75)^a^	35 (28, 50)	33 (21, 50)	0.171	35 (26, 50)
Age group (years)				
18–30	160	71	0.075	231
31–50	178	49		227
>50	97	40		137
Blood type				
A	124	40	0.037	164
B	118	61		179
O	133	35		168
AB	60	24		84
BMI				
<18.5	10	2	0.722	12
18.5–23.9	385	143		528
>23.9	40	15		55
Occupation				
Medical staff	308	149	0.768	457
Worker	77	61		138

a M (P25, P75): median (interquartile range).

### 3.2 Dynamics of positive rate of neutralizing antibodies after vaccination

**Figure 2:**
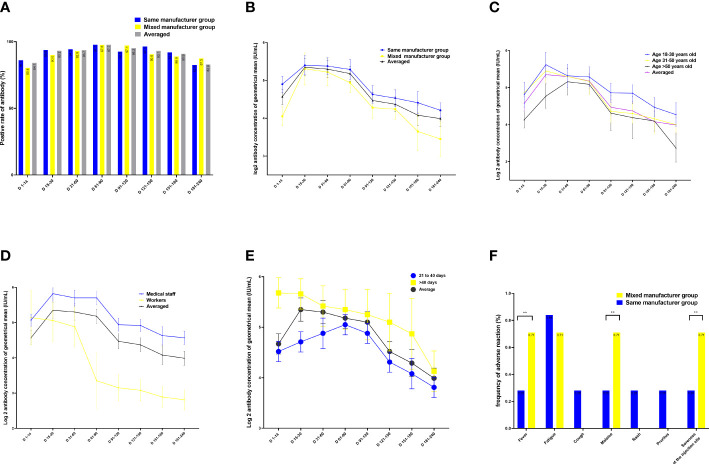
Quantitation of antibodies on days 1–240 following administration of the Chinese inactivated vaccine. **(A)** Positive rates of neutralizing antibodies. (**B–E**) Kinetics of neutralizing antibodies according to **(B)** vaccination type, **(C)** age, **(D)** occupation, and **(E)** sex. **(F)** Comparison of adverse effects between individuals vaccinated with vaccines from the same and different manufacturers for the two doses. Data are presented as mean (95% confidence interval [CI]) from the linear mixed-effects model adjusted for vaccine manufacturer, sex, blood type, age, occupation, and BMI. The log_2_-transformed level of neutralizing antibodies was used as the independent variable. BMI, body mass index; LSMD, least-square mean difference.

In both intervention and control groups, the positive rate of neutralizing IgG antibodies specific to the SARS-CoV-2 RBD slowly increased to 84.1% from 1 to 14 days, peaked at 97.7% from 60 to 90 days, and then slowly decreased and remained at 82.9% from 180 to 240 days after the second vaccination. There were no differences in the positive rate of neutralizing antibodies between the groups throughout the observation period (*p =* 0.891; **Figure 2A**).

### 3.3 Characteristics and influencing factors of neutralizing antibody production after vaccination

A linear regression analysis using a mixed-effect model showed that age, vaccination mode, occupation, interval between doses of vaccination, and vaccination duration were factors significantly associated with the levels of neutralizing antibodies (**Tables 1**–**3**; **Tables S1–S7**).

**Table 2:** Factors associated with the neutralization antibody concentration after the administration of the Chinese inactivated COVID-19 vaccine.

Factor	*n* (%)	B (95% CI)	*p*
Sex			
Men	146 (24.5)	Reference	
Women	449 (75.5)	0.028 (−0.22 to 0.27)	0.825
Age (years)			
Age	595	−0.02 (−0.03 to −0.015)	<0.001
18–30	231 (38.8)	Reference	
31–50	227 (38.2)	−0.31 (−0.5 to −0.1)	0.009
>50	137 (23.0)	−0.63 (−0.89 to −0.37)	<0.001
Blood type			
A	164 (27.6)	Reference	
B	179 (30.1)	0.1 (−0.2 to −0.3)	0.659
O	168 (28.2)	−0.2 (−0.4 to −0.1)	0.185
AB	84 (14.1)	0.1 (−0.2 to 0.4)	0.591
Vaccination type			
Same manufacturer	435 (73.1)	Reference	
Mixed manufacturer	160 (26.9)	−0.51 (−0.7 to −0.3)	<0.001
BMI (kg/m^2^)			
<18.5	16 (2.7)	Reference	
18.5–23.9	440 (73.9)	−0.48 (−1.2 to 0.2)	0.195
>23.9	139 (23.4)	−0.35 (−1.1 to 0.4)	0.379
Duration after vaccination (days)			
Mean days	595	−0.004 (−0.005 to −0.002)	<0.001
1–14	169 (28.4)	Reference	
15–30	62 (10.4)	0.66 (0.27–1.1)	0.001
31–60	51 (8.6)	0.57 (0.16–0.98)	0.007
61–90	131 (22.0)	0.58 (0.27–0.90)	<0.001
91–120	101 (17.0)	−0.12 (−0.5 to 0.2)	0.498
121–150	128 (21.5)	−0.21 (−0.5 to 0.1)	0.202
151–180	42 (7.1)	−0.54 (−1.1 to −0.1)	0.024
181–240	119 (20.0)	−0.7 (−1.0 to −0.3)	<0.001
Occupation			
Medical staff	457 (76.8)	Reference	
Worker	138 (23.2)	−1.0 (−1.5 to −0.55)	<0.001
Interval between doses of vaccination			
Mean days	595	0.015 (0.01–0.021)	<0.001
21–40	516	Reference	
>40	79	0.55 (0.25–0.84)	<0.001

**Table 3:** Factors associated with the levels of interferon-gamma after the administration of the Chinese inactivated COVID-19 vaccine.

Factor	*n* (%)	B (95% CI)	*p*
Sex			
Men	129 (25.1)	Reference	
Women	384 (74.9)	−0.08 (−0.2 to 0.04)	0.185
Age (years)			
Age	513	−0.005 (−0.008 to −0.001)	0.015
18–30	210 (40.9)	Reference	
31–50	207 (40.4)	0.1 (−0.1 to 0.1)	0.90
>50	96 (18.7)	−0.15 (−0.3 to −0.01)	0.03
Blood type			
A	143 (27.9)	Reference	
B	153 (29.8)	0.16 (0.04–0.28)	0.01
O	139 (27.1)	−0.01 (−0.1 to 0.1)	0.834
AB	78 (15.2)	0.03 (−0.1 to 0.2)	0.7
Vaccination type			
Same manufacturer	391 (76.2)	Reference	
Mixed manufacturer	122 (23.8)	0.02 (−0.09 to 0.1)	0.668
BMI (kg/m^2^)			
<18.5	12 (2.3)	Reference	
18.5–23.9	446 (86.9)	0.04 (−0.3 to 0.3)	0.806
>23.9	55 (10.7)	0.1 (−0.2 to 0.4)	0.514
Duration after vaccination (days)			
Mean days	513	−0.004 (−0.005 to 0.003)	0.105
1–14	26 (5.1)	Reference	
15–30	36 (7.0)	0.05 (−0.2 to 0.3)	0.71
31–60	39 (7.6)	0.07 (−0.2 to 0.3)	0.604
61–90	118 (23.0)	−0.2 (−0.4 to 0.1)	0.104
91–120	92 (17.9)	0.1 (−0.1 to 0.3)	0.322
121–150	82 (16.0)	0.06 (−0.2 to 0.3)	0.606
151–180	23 (4.5)	0.2 (−0.1 to 0.5)	0.177
181–240	97 (18.9)	0.2 (−0.1 to 0.4)	0.08
Occupation			
Medical staff	374 (72.9)	Reference	
Worker	139 (27.1)	−0.27 (−0.46 to −0.07)	0.008
Interval between doses of vaccination			
Mean days	513	−0.001 (−0.003 to 0.01)	0.107
21–40	434	Reference	
>40	79	0.074 (−0.01 to 0.15)	0.071

After adjusting for age, sex, occupation, blood type, interval between doses of vaccination, vaccination duration, and BMI, the highest geometric mean concentration (GMC) of neutralizing antibodies was 53.1 IU/mL in the same-manufacturer vaccination group at 15–30 days, which was slightly but significantly higher than the 44.6 IU/mL found in the mixed-manufacturer vaccination group (*p <* 0.001) (**Table 2**; **Figure 2B**). From days 31 to 240, there was a slight decrease in the GMC of neutralizing antibodies in both groups. At 240 days after vaccination, the GMC of neutralizing antibodies in the mixed-manufacturer vaccination group decreased to 8.5 IU/mL, which was less than the decrease detected in the same-manufacturer vaccination group (20.5 IU/mL) (*p <* 0.001). After the administration of the inactivated vaccine, the GMC of neutralizing antibodies decreased from 50.2 IU/mL on day 30 to 17.7 IU/mL (average decrease: 32.3%) and 13.3 IU/mL (average decrease: 25.9%) on days 180 and 240, respectively, representing an average monthly decrease of 10.1% (**Figure 2B**). Interestingly, the concentration of neutralizing antibodies decreased by an average of 0.004 IU (95% confidence interval [CI]: 0.002–0.005, *p <* 0.001) per day after vaccination (**Table 2**).

We identified age as a contributing factor; with increasing age, the antibody concentration generated was decreased, and the rate of decrease in the neutralizing antibody concentration was rapid (age 31–50 years vs. age 18–30 years, B = −0.31, 95% CI −0.5 to −0.1; *p =* 0.009; **Table 2**; **Figure 2C**). Occupation was another contributing factor, as the medical staff produced one-fold more neutralizing antibodies than the workers during the 1–240 days after vaccination (B = −1.0, 95% CI −1.5 to −0.55; *p <* 0.001; **Table 2**; **Figure 2D**). Women had 6.5 IU more neutralizing antibodies than men (B = 0.7, 95% CI 0.11–1.28; *p =* 0.021; least-square mean difference = 6.5, 95% CI 5.1–10.1, *p =* 0.019) 240 days after vaccination (**Table S1**). Participants with blood type A had lower levels of neutralizing antibodies than those with blood type B during 1–14 days after vaccination (least-square mean difference = 15.8, 95% CI 11.0–25.2; *p =* 0.044) (**Table S2**). Participants with an interval of <40 days between the doses of vaccination had lower levels of neutralizing antibodies than those with an interval of >40 days between the doses (B = 0.015 (0.01–0.021), 95% CI 0.01–0.021; *p <* 0.001) (**Figure 2E**; **Table 2**, and **Table S3**).

### 3.4 Characteristics of the reactogenicity after vaccination

Reactogenicity analysis was based on solicited adverse events in 425 and 143 participants from the control and intervention groups, respectively, 30 days after vaccination. In both groups, most of the adverse events were mild (n = 25, 81.1%) or moderate (n = 6, 18.9%) and self-limited. The most common adverse effect was fatigue (n = 4), followed by fever (n = 2), injection site pain (n = 4), malaise (n = 4), rash (n = 4), and pruritus (n = 3). However, the incidence of fever, injection site pain, and malaise in the mixed-manufacturer vaccination group was slightly but significantly (*p* < 0.01) higher (1/143, 0.71%) than in the same-manufacturer vaccination group (1/425, 0.28; **Figure 2F**).

### 3.5 Characteristics and factors influencing the cellular immune response after vaccination

**Figure 3:**
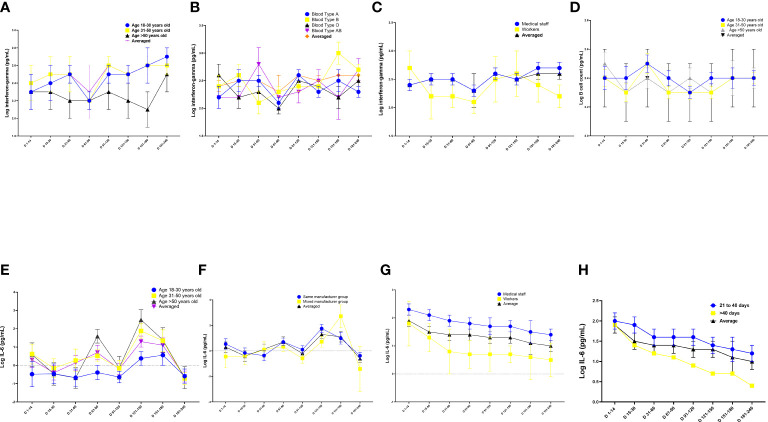
Quantitation of cell immune responses on days 1–240 following administration of the Chinese inactivated vaccine. **(A–C)** Kinetics of interferon-gamma levels according to **(A)** age, **(B)** blood type, and **(C)** occupation. **(D)** Kinetics of B-lymphocyte count according to age. **(E–G)** Kinetics of interferon (IL)-6 levels according to **(E)** age, **(F)** vaccine manufacturer, **(G)** occupation and **(H)** interval between doses of vaccination. Data were calculated based on a linear mixed-effects model adjusted for vaccine manufacturer, sex, age, blood type, occupation, and body mass index (BMI). The log-transformed level of interferon-gamma, CD4+ T-cell count, B-lymphocyte count, and IL-6 level were used as independent variables.

The multiple linear regression analysis revealed that participants aged >50 years had lower reactive IFN-γ levels and B-lymphocyte counts than those aged 18–30 years (**Tables 3**–**6**, **Figures 3A–G**; **Tables S8–S15**) (B = −0.15, *p =* 0.03; B = −0.04, *p =* 0.036, respectively) (**Tables 3**, **5**; **Figures 3A, D**). Conversely, participants aged >50 years had higher levels of reactive IL-6 than those aged 18–30 years (B = 1.0, 95% CI 0.6–1.5; *p <* 0.001) (**Table 6**; **Figure 3E**).

**Table 4:** Factors associated with CD4+ T-lymphocyte count after the administration of the Chinese inactivated COVID-19 vaccine.

Factor	*n* (%)	B (95% CI)	*p*
Sex			
Men	131 (24.1)	Reference	
Women	412 (75.9)	0.035 (−0.1 to 0.71)	0.055
Age (years)			
Age	543	−0.001 (−0.01 to 0.01)	0.666
18–30	215 (39.6)	Reference	
31–50	216 (39.8)	−0.02 (−0.03 to 0.03)	0.714
>50	112 (20.6)	0.007 (−0.03 to 0.05)	0.915
Blood type			
A	148 (27.3)	Reference	
B	161 (29.7)	0.008 (−0.03 to 0.04)	0.683
O	148 (27.3)	0.015 (−0.02 to 0.05)	0.432
AB	86 (15.8)	0.015 (−0.03 to 0.06)	0.519
Vaccination type			
Same manufacturer	413 (76.1)	Reference	
Mixed manufacturer	130 (23.9)	0.007 (−0.03 to 0.04)	0.669
BMI (kg/m^2^)			
<18.5	13 (2.4)	Reference	
18.5–23.9	465 (85.6)	−0.04 (−0.13 to 0.05)	0.373
>23.9	65 (12.0)	−0.06 (−0.16 to 0.04)	0.234
Duration after vaccination (days)			
Mean days	543	−0.004 (−0.005 to 0.003)	0.105
1–14	41 (7.6)	Reference	
15–30	53 (9.8)	0.66 (0.27–1.1)	0.883
31–60	44 (8.1)	0.57 (0.16–0.98)	0.67
61–90	118 (21.7)	0.58 (0.27–0.90)	0.81
91–120	95 (17.5)	−0.12 (−0.5 to 0.2)	0.463
121–150	76 (14.0)	−0.21 (−0.5 to 0.1)	0.283
151–180	20 (3.7)	−0.54 (−1.1 to −0.1)	0.803
181–240	96 (17.7)	−0.7 (−1.0 to −0.3)	0.843
Occupation			
Medical staff	405 (74.6)	Reference	
Worker	138 (25.4)	0.05 (−0.01 to 0.11)	0.113
Interval between doses of vaccination			
Mean days	543	0.001 (−0.001 to 0.002)	0.47
21–40	467	Reference	
>40	76	−0.014 (−0.05 to 0.03)	0.503

**Table 5:** Factors associated with B-lymphocyte count after the administration of the inactivated Chinese COVID-19 vaccine.

Factor	*n* (%)	B (95% CI)	*p*
Sex			
Men	131 (24.3)	Reference	
Women	408 (75.7)	0.034 (−0.01 to 0.1)	0.142
Age (years)			
Age	539	−0.001 (−0.01 to 0.01)	0.058
18–30	211 (39.1)	Reference	
31–50	216 (40.1)	−0.04 (−0.08 to −0.003)	0.036
>50	112 (20.8)	−0.04 (−0.09 to 0.01)	0.155
Blood type			
A	144 (26.7)	Reference	
B	160 (29.7)	−0.027 (−0.07 to 0.02)	0.258
O	148 (27.5)	0.02 (−0.03 to 0.07)	0.414
AB	87 (16.1)	0.04 (−0.02 to 0.09)	0.185
Vaccination type			
Same manufacturer	412 (76.4)	Reference	
Mixed manufacturer	127 (23.6)	−0.002 (−0.05 to 0.04)	0.918
BMI (kg/m^2^)			
<18.5	12 (2.2)	Reference	
18.5–23.9	463 (85.9)	−0.09 (−0.2 to 0.04)	0.932
>23.9	64 (11.9)	−0.07 (−0.2 to 0.05)	0.912
Duration after vaccination (days)			
Mean days	539	−0.004 (−0.005 to 0.003)	0.008
1–14	28 (5.2)	Reference	
15 to 30	36 (6.7)	−0.05 (−0.2 to 0.05)	0.291
31 to 60	38 (7.1)	0.04 (−0.1 to 0.1)	0.456
61–90	118 (21.9)	−0.1 (−0.1 to 0.1)	0.166
91–120	93 (17.3)	−0.09 (−0.5 to −0.1)	0.049
121–150	106 (19.7)	−0.09 (−0.5 to −0.1)	0.041
151–180	23 (4.3)	−0.04 (−0.2 to 0.1)	0.472
181–240	97 (18.0)	−0.1 (−1.0 to 0.1)	0.87
Occupation			
Medical staff	401 (74.4)	Reference	
Worker	138 (25.6)	0.05 (−0.01 to 0.11)	0.113
Interval between doses of vaccination			
Mean days	539	0.001 (−0.001 to 0.002)	0.716
21–40	76	Reference	
>40	463	0.02 (−0.03 to 0.07)	-0.465

**Table 6:** Factors associated with IL-6 level after the administration of the Chinese COVID-19 inactivated vaccine.

Factor	*n* (%)	B (95% CI)	*p*
Sex			
Men	129 (25.2)	Reference	
Women	383 (74.8)	0.2 (−0.2 to 0.6)	0.319
Age (years)			
Age	512	0.029 (0.016 to 0.042)	0.001
18–30	210 (41.0)	Reference	
31 to 50	206 (40.2)	0.62 (0.27 to 0.97)	0.001
>50	96 (18.8)	1.0 (0.6 to 1.5)	<0.001
Blood type			
A	143 (27.9)	Reference	
B	153 (29.9)	−0.04 (−0.44 to 0.35)	0.838
O	138 (26.9)	−0.15 (−0.6 to 0.25)	0.47
AB	78 (15.2)	−0.17 (−0.7 to 0.3)	0.485
Vaccination type			
Same manufacturer	391 (76.4)	Reference	
Mixed manufacturer	121 (23.6)	−0.44 (−0.8 to −0.1)	0.019
BMI (kg/m^2^)			
<18.5	12 (2.3)	Reference	
18.5–23.9	445 (86.9)	0.9 (−0.1 to 1.9)	0.08
>23.9	55 (10.8)	0.8 (−0.1 to 1.9)	0.17
Duration after vaccination (days)			
Mean days	512	−0.004 (−0.006 to 0.002)	0.001
1–14	27 (5.3)	Reference	
15–30	36 (7.0)	−0.6 (−1.5 to 0.2)	0.144
31–60	38 (7.4)	−0.7 (−1.5 to 0.2)	0.126
61–90	118 (23.0)	0.3 (−0.4 to 1.0)	0.439
91–120	92 (17.9)	−0.49 (−1.2 to 0.3)	0.198
121–150	82 (16.0)	1.0 (0.3 to 1.8)	0.009
151–180	23 (4.5)	0.81 (−0.1 to 1.8)	0.098
181–240	96 (18.8)	−0.9 (−1.6 to −0.1)	0.021
Occupation			
Medical staff	373 (72.9)	Reference	
Worker	139 (27.1)	−1.5 (−2.1 to −0.9)	<0.001
Interval between doses of vaccination			
Mean days	512	−0.01 (−0.014 to −0.003)	0.003
21–40	431	Reference	
>40	81	−0.37 (−0.63 to −0.1)	0.007

Participants with blood type A and workers had lower levels of IFN-γ than those with blood type B and medical staff, respectively (B = 0.16, 95% CI 0.04–0.28, *p =* 0.01 and B = −0.27, 95% CI −0.46 to −0.07, *p =* 0.008, respectively; **Table 3**; **Figures 3B, C**). Participants in the mixed-manufacturer vaccination group, those with an interval of >40 days between doses of vaccination, and workers produced lower levels of IL-6 than those in the same-manufacturer vaccination group, those with an interval of <40 days between doses of vaccination, and medical staff (B = −0.01, 95% CI −0.014 to −0.003, *p =* 0.003, B = −0.44, 95% CI −0.8 to −0.1, *p =* 0.019, and B = −1.5, 95% CI −2.1 to −0.9, *p <* 0.001, respectively; **Table 6**; **Figures 3F–H**). There were no changes in IFN-γ levels and CD4 T+ lymphocyte count on days 1 to 240 (B = 0.2, 95% CI −0.03 to 0.4, *p =* 0.09 and B = −0.004, 95% CI −0.005 to 0.003, *p =* 0.105; **Tables 3**, **4**) in participants stimulated with the SARS-CoV-2 S antigen. In contrast, we observed a decrease in the B-lymphocyte count and IL-6 level on days 1 to 240 in participants stimulated with the SARS-CoV-2 S antigen (B = −0.004, 95% CI −0.005 to −0.003; *p =* 0.008 and B = −0.004, 95% CI −0.006 to −0.002, *p =* 0.001, respectively; **Tables 5**, **6**; **Figures 3A, G**).

## 4 Discussion

To our knowledge, this study is the first to report that a heterologous Chinese COVID-19 inactivated vaccine administration schedule induced humoral and cellular immune responses in humans and that it was associated with an acceptable and manageable reactogenicity profile 240 days after vaccination. Factors that influence humoral and cellular immune responses were defined within 240 days in individuals vaccinated with an inactivated Chinese vaccine. Furthermore, this study was the first to explore the association between the blood type of participants and humoral and cellular immune responses within 240 days after the administration of a Chinese inactivated vaccine. The early response observed 30 days after the second dose showed a boost effect linked to the same manufacturer’s scheme. Immune cellular responses at 1–240 days after the second dose of vaccine also supported the same manufacturer’s approach. Immune responses to the heterologous vaccination schedule were within the range of those reported using homologous schedules. Neutralizing antibody levels were associated with a 33.89% decrease in anti-SARS-CoV-2 spike protein IgG standardized ELISA titers 180 days after the second vaccination (21), similar to our results, that is, a 32.3% decrease in neutralizing antibody levels in the following 180 days.

In this study, a higher frequency of adverse events reported by participants in the mixed-manufacturer vaccination group. Individuals vaccinated with different vaccines, those with blood type A, and workers had weaker humoral and cellular immune responses to vaccines than those vaccinated with the same vaccine, those with blood type B, and medical staff. Unfortunately, to date, occupation- and blood type-disaggregated data on cellular and humoral immunogenicity have not been reported by studies on COVID-19 vaccines.

Neutralizing antibody levels usually increase after the second administration of mixed vaccines (22, 23). However, in this study, the GMC in participants in the mixed-manufacturer group was lower than that in participants in the same-manufacturer group, which is perhaps due to the fact that vaccines from different manufacturers were still of the same type (both inactivated vaccines, but a protein or attenuated live vaccine). The profile of solicited adverse events in this study was consistent with that of a previous study (20).

Associations were observed between blood type and production of neutralizing antibodies and IFN-γ. Similarly, previous studies reported that patients with blood group A had an increased risk of infection with SARS-CoV-2, whereas those with blood group O were associated with a decreased risk (21, 24). Interestingly, individuals with blood type A produced lower amounts of IFN-γ at 180–240 days after vaccination and had lower neutralizing antibodies at 1–14 days than those with blood type B. This may explain why individuals with blood type A were more susceptible to SARS-CoV-2 infection than those with other blood types. However, this speculation requires validation using a larger patient cohort. The levels of neutralizing antibodies and IFN-γ were higher in medical staff 240 days after vaccination than in workers. This may be due to the increased exposure of medical staff to patients with COVID-19 compared to people in other occupations.

In this study, the older population produced lower levels of neutralizing antibodies, IFN-γ, B-lymphocytes, and IL-6, similar to that in previous studies (3, 25). The levels of IFN-γ and CD4+ T-lymphocytes were not altered in the participants for 240 days after vaccination, whereas the levels of neutralizing antibodies, B-lymphocytes, and IL-6 decreased with increasing time after vaccination. This finding suggests that cellular immune responses lasted longer than humoral responses in the vaccinated population. The levels of cellular immune molecules, such as IFN-γ and CD4+ T-lymphocytes, reflect the levels of effector molecules involved in the humoral immune response, such as memory B cells and neutralizing antibodies (26). Therefore, we speculate that the concentration of neutralizing antibodies may be increased more rapidly after the third or fourth dose of booster. We found that participants with a longer interval between vaccination doses (40–56 days) had higher levels of neutralizing antibodies than those with a shorter interval (21–40 days). This result indicates that by changing the interval between vaccinations with the inactivated vaccine from the current 3–8 weeks to 6–10 weeks (12), vaccinated people can produce higher levels of neutralizing antibody.

This study had limitations. The number of participants was relatively small, and at the time of the clinical study design, the administrators did not advocate the administration of vaccines from different manufacturers for separate doses. Therefore, we were able to collect data of a few individuals who had received mixed vaccinations at the time. Whether the immunogenic response observed in this study will translate to better efficacy and effectiveness—a fact that should be considered in strategic decisions about vaccination programs—is unknown. Second, the reported adverse events could also have been underestimated due to the small number of participants and short observation period. Third, because of limited resources, we could not perform pseudo-virus neutralization tests of neutralizing antibodies against COVID-19 (27). Therefore, we could not determine the level of neutralizing antibodies against viruses that cause breakthrough infection or severe infection. We only observed a trend of decreasing neutralizing antibodies after vaccination over time. In the future, we will include more participants after administering booster vaccines and conduct pseudo-virus neutralization tests to address these limitations.

In conclusion, this study is the first to evaluate robust humoral and cellular immune responses at 240 days in 595 participants after a second dose of inactivated vaccine in individuals primed with the first dose. Most participants rapidly developed neutralizing antibodies after two doses of the Chinese COVID-19 inactivated vaccine administered 3 weeks apart. The relationships between blood type-, age-, sex-, and occupation-related reactivities of neutralizing antibodies and cellular immune responses were demonstrated. Finally, individuals with certain occupations, including workers, especially older individuals and men, had low levels of antibodies and had weakened cellular immune responses following the second dose compared to their counterparts, suggesting that a longer gap between vaccine doses, which is a strategy in effect in some countries, should be re-evaluated, especially for more susceptible populations.

## Data availability statement

The original contributions presented in the study are included in the article/**Supplementary Material**. Further inquiries can be directed to the corresponding author.

## Ethics statement

The studies involving human participants were reviewed and approved by the ethics committee of Henan Provincial People’s Hospital. The patients/participants provided their written informed consent to participate in this study.

## Author contributions

YY and JX designed the study, analyzed the data, and wrote the manuscript. BM, GC, and ZW contributed to the collection and interpretation of the laboratory and clinical data. NJ, BW, QiZ, and JZ analyzed the immune data of the participants. YL, SW, and WY were involved in the project management and organizational work. BM and JZ collected data and YL reviewed the manuscript. All authors contributed to the article and approved the submitted version.
