# Supplementary material for: Characteristics of humoral and cellular responses to coronavirus disease 2019 (COVID-19) inactivated vaccine in central China: A prospective, multicenter, longitudinal study
Source: Front Immunol. 2023 Mar 3;14:1107866. doi: 10.3389/fimmu.2023.1107866 (PMC10020218; doi:10.3389/fimmu.2023.1107866)
Supplement: Supplementary file 1 [file DataSheet_1.doc]

Supplementary Material

# Supplementary Tables

**Supplementary Table 1**. Factors associated with the concentration of neutralizing antibodies after vaccination with the Chinese inactive COVID-19 vaccine.

| Factors | Days post-vaccination | | | | | | | | | | | |
| --- | --- | --- | --- | --- | --- | --- | --- | --- | --- | --- | --- | --- |
|  | 1–14 (n = 169) | | | 15–30 (n = 62) | | | 31–60 (n = 52) | | | 61–90 (n = 131) | | |
|  | *n* | B (95% CI) | *p* | *n* | B (95% CI) | *p* | *n* | B (95% CI) | *p* | *n* | B (95% CI) | *p* |
| Sex | | | | | | | | | |  |  |  |
| Men | 33 | Reference |  | 13 | Reference |  | 14 | Reference | 1 | 26 | Reference |  |
| Women | 136 | 0.34 (0.3–0.9) | 0.290 | 49 | 0.02 (0.74–0.77) | 0.970 | 38 | 0.3 (−0.5–1.1) | 0.440 | 105 | 0.78 (0.3–1.3) | 0.002 |
| Age (years) | | | | | | | | | |  |  |  |
| Age | 169 | −0.037 (0.02–0.06) | <0.001 | 62 | −0.026 (−0.05–0.003) | 0.030 | 52 | 0.01 (−0.03–0.02) | 0.99 | 131 | 0.01 (−0.02–0.01) | 0.440 |
| 18–30 | 22 | Reference |  | 19 | Reference |  | 21 | Reference | 1 | 36 | Reference |  |
| 31–50 | 65 | 0.02 (0.79–0.84) | 0.960 | 24 | 0.39 (0.39–1.17) | 0.340 | 17 | 0.2 (−0.6–0.9) | 0.810 | 55 | 0.2 (−0.3–0.7) | 0.400 |
| >50 | 82 | −0.39 (0.08–1.9) | 0.060 | 19 | 0.5 (0.3–1.3) | 0.230 | 14 | 0.3 (−1.1–0.5) | 0.740 | 40 | 0.03 (−0.5–0.6) | 0.900 |
| Blood type | | | | | | | | | |  |  |  |
| A | 34 | Reference |  | 16 | Reference |  | 23 | Reference |  | 29 | Reference |  |
| B | 61 | 0.71 (0.03–1.4) | 0.043 | 15 | 0.51 (0.4–1.4) | 0.270 | 11 | 0.5 (−0.4–1.4) | 0.620 | 37 | 0.2 (−0.7–0.4) | 0.600 |
| O | 35 | 0.29 (0.45–1.0) | 0.440 | 22 | 0.52 (0.3–1.3) | 0.200 | 12 | 0.1 (−0.7–1.0) | 1.140 | 42 | 0.5 (0.04–1.0) | 0.070 |
| AB | 39 | 0.83 (0.09–1.6) | 0.037 | 9 | 0.4 (−0.6–1.3) | 0.450 | 6 | 0.1 (−1–1.2) | 1.120 | 23 | 0.2 (−0.8–0.4) | 0.500 |
| Vaccination type | | | | | | | | | |  |  |  |
| Same manufacturer | 112 | Reference |  | 51 | Reference |  | 38 | Reference | - | 89 | Reference |  |
| Mixed manufacturer | 57 | 0.88 (0.37–1.4) | 0.007 | 11 | 0.1 (−0.8–0.9) | 0.990 | 14 | 0.4 (−1–0.4) | 0.350 | 42 | 0.5 (0.04–0.9) | 0.032 |
| BMI (kg/m^2^) |  | | | | | | | | | | | |
| <18.5 | 2 | Reference |  | 3 | Reference |  | 2 | Reference |  | 0 |  |  |
| 18.5–23.9 | 110 | 0.024 (−3.9–0.8) | 0.173 | 48 | −0.6 (−2.1–1.0) | 0.487 | 41 | −0.08 (−1.9–1.8) | 0.93 | 109 | Reference |  |
| >23.9 | 57 | −1.59 (−3.9 to 0.7) | 0.187 | 11 | −0.8 (−2.5–1.0) | 0.387 | 9 | 0.3 (−1.6–2.3) | 0.74 | 22 | −0.12 (−0.6–0.4) | 0.663 |
| Occupation |  |  |  |  |  |  |  |  |  |  |  |  |
| Medical staff | 139 | Reference |  | 33 | Reference |  | 27 | Reference |  | 71 | Reference |  |
| Worker | 30 | 1.64 (−0.2–3.4) | 0.075 | 29 | 1.1 (−0.74–2.9) | 0.246 | 25 | 0.6 (−0.7–2.0) | 0.381 | 60 | 0.29 (−0.7–1.2) | 0.552 |
| Interval between two doses of vaccination. | | | | | | | | | | | | |
| 21–40 days | 265 | Reference |  | 98 | Reference |  | 74 | Reference |  | 241 | Reference |  |
| >40 days | 20 | 1.05 (0.4–1.8) | 0.003 | 25 | 0.75 (0.13–1.4) | 0.017 | 16 | 0.14 (−0.6–0.9 | 0.721 | 31 | 0.57 (0.1–1.1) | 0.031 |

**Supplementary Table 1.** Factors associated with the concentration of neutralizing antibodies after vaccination with the Chinese inactive COVID-19 vaccine (continued)

| Factors | Days post-vaccination | | | | | | | | | | | |
| --- | --- | --- | --- | --- | --- | --- | --- | --- | --- | --- | --- | --- |
|  | 91–120 (*n* = 101) | | | 121–150 (*n* = 128) | | | 151–180 (*n* = 42) | | | 181–240 (*n* = 119) | | |
|  | *n* | B (95% CI) | *p* | *n* | B (95% CI) | *p* | *n* | B (95% CI) | *p* | *n* | B (95% CI) | *p* |
| Sex | | | | | | | | | |  |  |  |
| Men | 16 | Reference |  | 22 | Reference |  | 11 | Reference | 0.100 | 48 | Reference |  |
| Women | 85 | 0.11 (−0.74–0.52) | 0.730 | 106 | 0.15 (−0.82–0.52) | 0.660 | 31 | 0.7 (−0.1–1.6) |  | 71 | 0.7 (0.11–1.28) | 0.021 |
| Age (years) | | | | | | | | | | | | |
| Age | 101 | 0.01 (−0.03–0.01) | 0.394 | 128 | −0.031 (0.01–0.05) | 0.007 | 42 | 0.02 (−0.02–0.05) | 0.291 | 119 | −0.03 (0.01–0.06) | 0.041 |
| 18–30 | 65 | Reference |  | 39 | Reference |  | 23 | Reference |  | 45 | Reference |  |
| 31–50 | 27 | −0.52 (−1.1–0.1) | 0.046 | 43 | −0.62 (−1.2–0.10) | 0.024 | 13 | 0.5 (0.4–1.4) | 0.250 | 60 | −0.7 (0.1–1.3) | 0.025 |
| >50 | 9 | 0.31 (−1.1–0.52) | 0.470 | 46 | −0.59 (−1.2–0.01) | 0.044 | 6 | 0.6 (−0.6–1.8) | 0.350 | 14 | −1 (0.03–2.1) | 0.044 |
| Blood type | | | | | | | | | | | | |
| A | 31 | Reference |  | 36 | Reference |  | 14 | Reference |  | 36 | Reference |  |
| B | 27 | 0.41 (−1.0–0.2) | 0.180 | 39 | 0.1 (−0.6–0.8) | 0.770 | 12 | 0.7 (−2.1–0.7) | 0.150 | 38 | 0.01 (−0.7–0.7) | 0.975 |
| O | 29 | 0.32 (−0.9–0.26) | 0.280 | 29 | 0.5 (−1.3–0.3) | 0.210 | 12 | 0.3 (−1.3–0.6) | 0.470 | 32 | 0.67 (−1.4–0.1) | 0.077 |
| AB | 14 | 0.67 (−1.4–0.07) | 0.075 | 24 | 0.11 (−1.1–0.9) | 0.830 | 4 | 0.71 (−2.2–0.7) | 0.330 | 13 | 0.23 (−0.7–1.2) | 0.630 |
| Vaccination type | | | | | | | | | | | | |
| Same manufacturer | 67 | Reference |  | 91 | Reference |  | 31 | Reference | 0.19 | 111 | Reference |  |
| Mixed manufacturer | 34 | 0.31 (−0.8–0.2) | 0.210 | 37 | 0.47 (−1.1–0.14) | 0.130 | 11 | 0.6 (−1.6–0.3) |  | 8 | 1.2 (−3.3–0.7) | 0.200 |
| BMI (kg/m^2^) |  | | | | | | | | | | | |
| <18.5 | 5 | Reference |  | 2 | Reference |  | 0 |  |  | 2 | Reference |  |
| 18.5–23.9 | 89 | −0.73 (−1.8–0.35) | 0.185 | 105 | 0.47 (−1.3–2.3) | 0.607 | 40 | Reference |  | 101 | 0.21 (−1.7–2.1) | 0.830 |
| >23.9 | 7 | −1.1 (−2.5–0.32) | 0.131 | 21 | 0.25 (−1.6–2.1) | 0.794 | 2 | 0.51 (−1.5–2.5) | 0.623 | 16 | 0.89 (−1.1–2.9) | 0.390 |
| Occupation |  | | | | | | | | | | | |
| Medical staff | 79 | Reference |  | 96 | Reference |  | 20 | Reference |  | 68 | Reference |  |
| Workers | 22 | 1.6 (−0.1–3.3) | 0.051 | 32 | −2.6 (−4.4–−0.1) | 0.005 | 23 | −0.51 (−2.3–1.3) | 0.579 | 51 | −1.2 (−2–−0.4) | 0.004 |
| Interval between two doses of vaccination. | | | | | | | | | | | | |
| 21–40 days | 125 | Reference |  | 27 | Reference |  | 61 | Reference |  | 129 | Reference |  |
| >40 days | 14 | −0.1 (−0.8 to 0.6) | 0.81 | 242 | −0.7 (−1.2 to −0.1) | 0.016 | 9 | 1.8 (0.9–2.8) | 0.001 | 12 | −0.17 (−1 to 0.6) | 0.68 |

Effects were evaluated using a linear mixed-effects model adjusted for vaccine manufacturer, sex, age, occupation, blood type, interval between two doses of vaccination, duration after vaccination, and BMI. The log2-transformed level of neutralizing antibody was the independent variable.

Abbreviations: BMI: body mass index; CI: confidence interval.

**Supplementary Table** **2.** Kinetics of the concentration of neutralizing antibodies^a^ after vaccination according to the type of blood after vaccination with the Chinese inactive COVID-19 vaccine.

| Factors | Days post-vaccination | | | | | | | | | |
| --- | --- | --- | --- | --- | --- | --- | --- | --- | --- | --- |
|  | 1–14 | 15–30 | 31–60 | 61–90 | 91–120 | 121–150 | 151–180 | 181–240 | F | *p* |
| Average | 33.3 ± 1.1 | 50.2 ± 1.2 | 41.3 ± 1.2 | 37.5 ± 1.1 | 29.1 ± 1.1 | 26.4 ± 1.1 | 17.7 ± 1.2 | 13.3 ± 1.4 | 3.47 | 0.001 |
| A | 26.1 ± 1.2 | 39.5 ± 1.3 | 43.0 ± 1.2 | 44.0 ± 1.2 | 37.0 ± 1.2 | 28.6 ± 1.2 | 25.5 ± 1.4 | 14.2 ± 1.4 | 2.739 | 0.009 |
| B | 41.9 ± 1.2 | 56.1 ± 1.3 | 30.8 ± 1.3 | 38.9 ± 1.2 | 27.9 ± 1.2 | 30.9 ± 1.3 | 14.9 ± 1.3 | 14.4 ± 1.3 | 2.310 | 0.038 |
| O | 31.9 ± 1.2 | 56.5 ± 1.2 | 47.2 ± 1.3 | 30.7 ± 1.1 | 29.8 ± 1.2 | 20.4 ± 1.2 | 18.6 ± 1.3 | 8.9 ± 1.4 | 3.387 | 0.002 |
| AB | 35.2 ± 1.2 | 50.9 ± 1.4 | 46.5 ± 1.4 | 37.3 ± 1.2 | 23.3 ± 1.3 | 26.7 ± 1.1 | 13.4 ± 1.5 | 17.1 ± 1.5 | 3.681 | 0.002 |
| LSMD (95% CI) | 15.8 (11.0–25.2) ^b^, ^[[1]](#footnote-1)^.91 (3.5,13.9) ^c^ | - | - | - | - | - | - | - |  |  |
| *F* | 1.884 | 0.100 | 0.580 | 0.867 | 1.150 | 0.417 | 0.797 | 1.287 |  |  |
| *p* | 0.044^b^, 0.037^c^ | 0.446 | 0.590 | 0.306 | 0.299 | 0.832 | 0.816 | 0.623 |  |  |

^a^Effects were evaluated using a linear mixed-effects model adjusted for vaccine manufacturer, sex, age, occupation, interval between two doses of vaccination, duration after vaccination, and BMI. The log2-transformed level of neutralizing antibody was the independent variable.

^b^Blood type B and AB vs. type A.

^c^Blood type AB vs. type A.

Abbreviations: CI: confidence interval; LSMD: least-square mean difference; -: not available.**Supplementary Table 3.** Kinetics of the concentration of neutralizing antibodies^a^ after vaccination according to the interval between two doses of vaccination with the Chinese inactive COVID-19 vaccine.

| Factors | Days post-vaccination | | | | | | | | | |
| --- | --- | --- | --- | --- | --- | --- | --- | --- | --- | --- |
|  | 1–14 | 15–30 | 31–60 | 61–90 | 91–120 | 121–150 | 151–180 | 181–240 | *F* | *p* |
| Average | 33.3 ± 1.1 | 50.2 ± 1.2 | 41.3 ± 1.2 | 37.5 ± 1.1 | 29.1 ± 1.1 | 26.4 ± 1.1 | 17.7 ± 1.2 | 13.3 ± 1.4 | 3.47 | 0.001 |
| 21–40 days | 24.5 ± 1.1 | 26.2 ± 1.2 | 35.5 ± 1.2 | 34.3 ±1.2 | 21.1 ± 1.1 | 19.7 ± 1.3 | 16.3 ± 1.2 | 12.3 ± 1.3 | 1.799 | 0.01 |
| >40 days | 51.3 ± 1.3 | 50.6 ± 1.3 | 42.2 ± 1.3 | 41.3 ± 1.3 | 40.8 ± 1.2 | 34.3 ± 1.2 | 29.9 ± 1.4 | 16.3 ± 1.3 | 4.543 | <0.001 |
| LSMD (95% CI) | 26.8 ± 1.3 | 24.4 ± 1.3 | 6.7 ± 1.3 | 7.0 ± 1.2 | 21.1 ± 1.2 | 13.2 ± 1.3 | 13.6 ± 1.4 | 4.0 ± 1.3 |  |  |
| *t* | 3.9 | 3.27 | 0.239 | 1.335 | 0.969 | 0.104 | 0.224 | 1.213 |  |  |
| *p* | 0.001 | 0.005 | 0.503 | 0.399 | 0.248 | 0.235 | 0.183 | 0.878 |  |  |

^a^Values were evaluated using a mixed line effect model adjusted for vaccine manufacturer, sex, age, blood type, interval between two doses of vaccination, duration after vaccination, and occupation. Log2 amount of neutralizing antibody was the independent variable.

Abbreviations: BMI: body mass index; CI: confidence interval; LSMD: least-square mean difference; -: not available.

**Supplementary Table 4.** Kinetics of the concentration of neutralization antibodies^a^ after vaccination according to the vaccine manufacturer after vaccination with the Chinese inactive COVID-19 vaccine.

| Factors | Days post-vaccination | | | | | | | | | |
| --- | --- | --- | --- | --- | --- | --- | --- | --- | --- | --- |
|  | 1–14 | 15–30 | 31–60 | 61–90 | 91–120 | 121–150 | 151–180 | 181–240 | *F* | *p* |
| Average | 33.3 ± 1.1 | 50.2 ± 1.2 | 41.3 ± 1.2 | 37.5 ± 1.1 | 29.1 ± 1.1 | 26.4 ± 1.1 | 17.7 ± 1.2 | 13.3 ± 1.4 | 3.47 | 0.001 |
| Same manufacturer | 38.1 ± 3.1 | 53.1 ± 2.5 | 46.5 ± 2.5 | 45.3 ± 2.3 | 32.4 ± 1.2 | 31.0 ± 1.2 | 22.1 ± 1.3 | 20.5 ± 1.1 | 15.9 | 0.001 |
| Mixed manufacturer | 20.9 ± 3.0 | 44.6 ± 2.9 | 36.5 ± 2.2 | 31.8 ± 2.3 | 25.9 ± 1.1 | 22.4 ± 1.2 | 14.1 ± 1.2 | 8.5 ± 1.7 | 6.98 | 0.001 |
| LSMD (95% CI) | 7.2 (5.3–9.6) | 8.5 (7.0–16.2) | 10.0 (5.1–13.1) | 13.5 (10.5–19.1) | 6.5 (5.6–11.7) | 8.6 (8.0–11.1) | 8.0 (6.4–13.1) | 12.0 (7.2–19.5) |  |  |
| *t* | 3.796 | 0.558 | 4.036 | 3.603 | 2.741 | 1.549 | 1.533 | 2.403 |  |  |
| *p* | 0.001 | 0.58 | 0.35 | 0.032 | 0.21 | 0.13 | 0.19 | 0.212 |  |  |

^a^Effects were calculated and based on a linear mixed-effects model adjusted by age, sex, blood type, occupation, interval between two doses of vaccination, duration after vaccination, and body mass index. The log2-transformed level of neutralizing antibody was the independent variable.

Abbreviations: CI: confidence interval; LSMD: least-square mean difference.

**Supplementary Table 5.** Kinetics of the concentration of neutralization antibodies^a^ after vaccination according to sex after vaccination with the Chinese inactive COVID-19 vaccine.

| Factors | Days post-vaccination | | | | | | | | | |
| --- | --- | --- | --- | --- | --- | --- | --- | --- | --- | --- |
|  | 1–14 | 15–30 | 31–60 | 61–90 | 91–120 | 121–150 | 151–180 | 181–240 | *F* | *p* |
| Average | 33.3 ± 1.1 | 50.2 ± 1.2 | 41.3 ± 1.2 | 37.5 ± 1.1 | 29.1 ± 1.1 | 26.4 ± 1.1 | 17.7 ± 1.2 | 13.3 ± 1.4 | 3.470 | 0.001 |
| Men | 34.9 ± 1.2 | 42.6 ± 1.3 | 36.9 ± 1.3 | 31.6 ± 1.1 | 28.1 ± 1.2 | 25.1 ± 1.3 | 12.9 ± 1.3 | 10.4 ± 1.4 | 3.230 | 0.003 |
| Women | 29.9 ± 1.1 | 50.5 ± 1.2 | 48.1 ± 1.2 | 47.5 ± 1.2 | 30.3 ± 1.1 | 27.3 ± 1.1 | 21.1 ± 1.2 | 16.9 ± 1.3 | 2.270 | 0.027 |
| LSMD (95% CI) | 5.0 (4.8–14) | 7.9 (4.7–13.7) | 11.2 (7.8–12.1) | 15.9 (7.2–18.4） | 2.2 (0.7–1.7) | 2.2 (1.5–2.8） | 8.2 (6.8–12.2) | 6.5 (5.1–10.1) |  |  |
| *t* | 1.143 | 0.1 | 0.599 | 3.16 | 0.587 | 0.46 | 1.257 | 2.212 |  |  |
| *p* | 0.255 | 0.967 | 0.444 | 0.004 | 0.731 | 0.663 | 0.141 | 0.019 |  |  |

^a^Effects were calculated and based on a linear mixed-effects model adjusted by vaccine manufacturers, age, blood type, occupation, interval between two doses of vaccination, duration after vaccination, and body mass index. The log2-transformed level of neutralizing antibody was the independent variable.

Abbreviations: CI: confidence interval; LSMD: least-square mean difference.

**Supplementary Table 6.** Kinetics of the concentration of neutralization antibodies^a^ after vaccination according to age after vaccination with the Chinese inactive COVID-19 vaccine.

| Factors | Days post-vaccination | | | | | | | | | |
| --- | --- | --- | --- | --- | --- | --- | --- | --- | --- | --- |
|  | 1–14 | 15–30 | 31–60 | 61–90 | 91–120 | 121–150 | 151–180 | 181–240 | *F* | *p* |
| Average | 33.3 ± 1.1 | 50.2 ± 1.2 | 41.3 ± 1.2 | 37.5 ± 1.1 | 29.1 ± 1.1 | 26.4 ± 1.1 | 17.7 ± 1.2 | 13.3 ± 1.4 | 3.47 | 0.001 |
| 18–30 years | 37.8 ± 1.3 | 66.7 ± 1.3 | 52.6 ± 1.2 | 45.7 ± 1.2 | 39.6 ± 1.1 | 37.3 ± 1.3 | 24.8 ± 1.2 | 19.3 ± 1.3 | 1.799 | 0.01 |
| 31–50 years | 37.0 ± 1.2 | 58.4 ± 1.3 | 47.5 ± 1.3 | 45.8 ± 1.1 | 27.2 ± 1.2 | 26.7 ± 1.2 | 19.3 ± 1.4 | 12.3 ± 1.3 | 4.543 | <0.001 |
| >50 years | 22.5 ± 1.1 | 37.2 ± 1.2 | 37.7 ± 1.3 | 36.5 ± 1.2 | 26.2 ± 1.3 | 20.5 ± 1.2 | 15.4 ± 1.5 | 11.3 ± 1.5 | 2.504 | 0.026 |
| LSMD (95% CI) | 15.3 (4.6–17.1) | 29.5 (9.1–31.9) | - | - | - | 8.9 (8.1–13.6) | - | 8.0 (7.1–12.5) |  |  |
| *F* | 3.801 | 2.474 | 0.239 | 1.335 | 0.969 | 3.923 | 0.224 | 2.710 |  |  |
| *p* | 0.001 | 0.019^b^ | 0.754 | 0.599 | 0.138 | 0.013^c^ | 0.347 | 0.028^c^ |  |  |

^a^Effects were calculated and based on a linear mixed effects model adjusted by vaccine manufacturers, sex, body mass index, occupation, interval between two doses of vaccination, duration after vaccination, and type of blood. The log2-transformed level of neutralizing antibody was the independent variable.

^b^Age: >50 years vs. 31–50 years.

^c^Age: >50 years vs. 18–30 years.

Abbreviations: CI: confidence interval; LSMD: least-square mean difference; -: not available.

**Supplementary Table 7.** Kinetics of the concentration of neutralization antibodies^a^ after vaccination according to occupation after vaccination with the Chinese inactive COVID-19 vaccine.

| Factors | Days post-vaccination | | | | | | | | | |
| --- | --- | --- | --- | --- | --- | --- | --- | --- | --- | --- |
|  | 1–14 | 15–30 | 31–60 | 6–90 | 91–120 | 121–150 | 151–180 | 181–240 | *F* | *p* |
| Average | 33.3 ± 1.1 | 50.2 ± 1.2 | 41.3 ± 1.2 | 37.5 ± 1.1 | 29.1 ± 1.1 | 26.4 ± 1.1 | 17.7 ± 1.2 | 13.3 ± 1.4 | 3.470 | 0.001 |
| Medical staff | 33.6 ± 1.1 | 58.9 ± 1.2 | 53.8 ± 1.2 | 46.9 ± 1.1 | 33.8 ± 1.1 | 28.7 ± 1.1 | 25.3 ± 1.2 | 23.2 ± 1.1 | 14.480 | <0.001 |
| Workers | 27.5 ± 1.7 | 28.4 ± 1.4 | 27.6 ± 1.5 | 19.7 ± 1.9 | 11.2 ± 1.9 | 8.9 ± 1.7 | 7.4 ± 1.9 | 6.3 ± 1.3 | 4.085 | 0.005 |
| LSMD (95% CI) | 3.3 (1.2–13.7) | 30.5 (21.8–36.8) | 26.2 (7.3–39.4) | 27.2 (14.9–36.8) | 22.6 (13.8–39.4) | 19.8 (12.8–34.3) | 17.9 (13.8–29.8) | 16.9 (13.9–18.1) |  |  |
| *t* | 0.200 | 3.080 | 1.490 | 1.350 | 1.600 | 2.260 | 1.299 | 4.010 |  |  |
| *p* | 0.840 | 0.002 | 0.137 | 0.178 | 0.110 | 0.026 | 0.194 | <0.001 |  |  |

^a^Effects were calculated and based on a linear mixed-effects model adjusted by vaccine manufacturers, blood type, sex, age, interval between two doses of vaccination, duration after vaccination, and body mass index. The log2-transformed level of neutralizing antibody was the independent variable.

Abbreviations: CI: confidence interval; LSMD: least-square mean difference.

**Supplementary Table 8.** Kinetics of the level of interferon gamma^a^ according to age after vaccination with the Chinese inactive COVID-19 vaccine.

| Factors | Days post-vaccination | | | | | | | | | |
| --- | --- | --- | --- | --- | --- | --- | --- | --- | --- | --- |
|  | 1–14 | 15–30 | 31–60 | 61–90 | 91–120 | 121–150 | 151–180 | 181–240 | *F* | *p* |
| Average | 2.4 ± 0.1 | 2.5 ± 0.1 | 2.5 ± 0.1 | 2.3 ± 0.3 | 2.6 ± 0.1 | 2.5 ± 0.1 | 2.7 ± 0.1 | 2.7 ± 0.1 | 5.360 | <0.001 |
| 18–30 years | 2.3 ± 0.2 | 2.4 ± 0.2 | 2.5 ± 0.1 | 2.2 ± 0.1 | 2.5 ± 0.1 | 2.5 ± 0.1 | 2.6 ± 0.2 | 2.7 ± 0.1 | 1.983 | 0.059 |
| 31–50 years | 2.4 ± 0.2 | 2.5 ± 0.2 | 2.5 ± 0.2 | 2.2 ± 0.1 | 2.6 ± 0.1 | 2.5 ± 0.1 | 2.6 ± 0.2 | 2.6 ± 0.1 | 2.256 | 0.031 |
| >50 years | 2.3 ± 0.2 | 2.3 ± 0.2 | 2.2 ± 0.2 | 2.2 ± 0.1 | 2.3 ± 0.2 | 2.2 ± 0.2 | 2.1 ± 0.5 | 2.5 ± 0.2 | 0.479 | 0.848 |
| LSMD  (95% CI) | - | - | - | - | - | - | - | - |  |  |
| *F* | 0.190 | 0.433 | 0.924 | 0.017 | 0.536 | 1.818 | 0.197 | 0.612 |  |  |
| *p* | 0.910 | 0.526 | 0.106 | 0.956 | 0.213 | 0.145 | 0.326 | 0.364 |  |  |

^a^Effects were calculated and based on a linear mixed-effects model adjusted by manufacturers, occupations, sex, blood type, interval between two doses of vaccination, duration after vaccination, and body mass index. The log-transformed level of interferon-gamma was the independent variable.

Abbreviations: CI: confidence interval; LSMD: least-square mean difference.

**Supplementary Table 9.** Kinetics of the level of interferon-gamma^a^ among blood types after vaccination with the Chinese inactive COVID-19 vaccine.

| Factors | Days post-vaccination | | | | | | | | | |
| --- | --- | --- | --- | --- | --- | --- | --- | --- | --- | --- |
|  | 1–14 | 15–30 | 31–60 | 61–90 | 91–120 | 121–150 | 151–180 | 181–240 | *F* | *p* |
| Average | 2.4 ± 0.1 | 2.5 ± 0.1 | 2.5 ± 0.1 | 2.3 ± 0.3 | 2.6 ± 0.1 | 2.5 ± 0.1 | 2.7 ± 0.1 | 2.7 ± 0.1 | 5.36 | <0.001 |
| A | 2.2 ± 0.2 | 2.5 ± 0.2 | 2.5 ± 0.1 | 2.1 ± 0.1 | 2.6 ± 0.1 | 2.3 ± 0.1 | 2.5 ± 0.2 | 2.3 ± 0.1 | 1.864 | 0.08 |
| B | 2.4 ± 0.2 | 2.6 ± 0.2 | 2.1 ± 0.2 | 2.3 ± 0.1 | 2.4 ± 0.1 | 2.4 ± 0.1 | 3.0 ± 0.2 | 2.7 ± 0.1 | 2.766 | 0.01 |
| O | 2.6 ± 0.2 | 2.2 ± 0.2 | 2.3 ± 0.2 | 2.0 ± 0.1 | 2.5 ± 0.1 | 2.4 ± 0.1 | 2.2 ± 0.2 | 2.5 ± 0.1 | 3.307 | 0.003 |
| AB | 2.2 ± 0.2 | 2.2 ± 0.2 | 2.8 ± 0.3 | 2.2 ± 0.1 | 2.3 ± 0.2 | 2.5 ± 0.2 | 2.2 ± 0.4 | 2. 7± 0.2 | 1.887 | 0.085 |
| LSMD (95% CI) | - | - | - | - | - | - | - | 0.4 (0.2–0.7) ^b^ |  |  |
| *F* | 1.029 | 1.415 | 1.547 | 3.765 | 0.634 | 0.273 | 1.544 | 3.052 |  |  |
| *p* | 0.279 | 0.663 | 0.079 | 0.078 | 0.484 | 0.482 | 0.066 | 0.002^a^ |  |  |

^a^Effects were calculated and based on a linear mixed-effects model adjusted by manufacturers, occupations, sex, age, interval between two doses of vaccination, duration after vaccination, and body mass index. The log-transformed level of interferon-gamma was the independent variable.

^b^Blood type B vs. blood type A.

Abbreviations: CI: confidence interval; LSMD: least-square mean difference.

**Supplementary Table 10.** Kinetics of the amount of interferon-gamma^a^ according to occupation after vaccination with the inactive Chinese COVID-19 vaccine.

| Factors | Days post-vaccination | | | | | | | | | |
| --- | --- | --- | --- | --- | --- | --- | --- | --- | --- | --- |
|  | 1–14 | 15–30 | 31–60 | 61–90 | 91–120 | 121–150 | 151–180 | 181–240 | *F* | *p* |
| Average | 2.4 ± 0.1 | 2.5 ± 0.1 | 2.5 ± 0.1 | 2.3 ± 0.3 | 2.6 ± 0.1 | 2.5 ± 0.1 | 2.7 ± 0.1 | 2.7 ± 0.1 | 5.360 | <0.001 |
| Medical staff | 2.4 ± 0.1 | 2.5 ± 0.1 | 2.5 ± 0.1 | 2.3 ± 0.1 | 2.6 ± 0.1 | 2.5 ± 0.1 | 2.7 ± 0.1 | 2.7 ± 0.1 | 6.030 | <0.001 |
| Workers | 2.7 ± 0.3 | 2.2 ± 0.4 | 2.2 ± 0.2 | 2.1 ± 0.2 | 2.5 ± 0.4 | 2.6 ± 0.4 | 2.4 ± 0.3 | 2.2 ± 0.2 | 0.556 | 0.785 |
| LSMD  (95% CI) | - | - | - | - | - | - | - | 0.5 (0.2–0.8) |  |  |
| *t* | 0.876 | 1.615 | 0.829 | 0.498 | 0.055 | 0.346 | 0.640 | 3.726 |  |  |
| *p* | 0.346 | 0.516 | 0.194 | 0.343 | 0.964 | 0.830 | 0.211 | 0.002 |  |  |

^a^Effects were calculated and based on a linear mixed-effects model adjusted by manufacturers, age, sex, blood type, interval between two doses of vaccination, duration after vaccination, and body mass index. The log-transformed level of interferon-gamma was the independent variable.

Abbreviations: CI: confidence interval; LSMD: least-square mean difference.

**Supplementary Table 11.** Kinetics of B lymphocyte^a^ count according to age after vaccination of the Chinese inactive COVID-19 vaccine.

| Factors | Days post-vaccination | | | | | | | | | |
| --- | --- | --- | --- | --- | --- | --- | --- | --- | --- | --- |
|  | 1–14 | 15–30 | 31–60 | 61–90 | 91–120 | 121–150 | 151–180 | 181–240 | *F* | *p* |
| Average | 2.4 ± 0.2 | 2.3 ± 0.2 | 2.4 ± 0.2 | 2.3 ± 0.2 | 2.3 ± 0.2 | 2.3 ± 0.2 | 2.4 ± 0.2 | 2.4 ± 0.2 | 2.781 | 0.008 |
| 18–30 years | 2.4 ± 0.08 | 2.4 ± 0.06 | 2.5 ± 0.05 | 2.4 ± 0.04 | 2.3 ± 0.04 | 2.4 ± 0.04 | 2.4 ± 0.07 | 2.4 ± 0.05 | 1.503 | 0.168 |
| 31–50 years | 2.4 ± 0.06 | 2.3 ± 0.06 | 2.5 ± 0.07 | 2.3 ± 0.04 | 2.3 ± 0.05 | 2.3 ± 0.04 | 2.4 ± 0.07 | 2.4 ± 0.04 | 2.041 | 0.051 |
| >50 years | 2.5 ± 0.08 | 2.3 ± 0.07 | 2.4 ± 0.07 | 2.3 ± 0.04 | 2.4 ± 0.07 | 2.3 ± 0.04 | 2.4 ± 0.12 | 2.4 ± 0.07 | 0.797 | 0.592 |
| LSMD (95% CI) | - | - | - | - | - | - | - | - |  |  |
| *F* | 0.526 | 0.384 | 0.883 | 2.806 | 1.539 | 0.887 | 0.086 | 0.024 |  |  |
| *p* | 0.903 | 0.617 | 0.188 | 0.065 | 0.361 | 0.338 | 0.864 | 0.441 |  |  |

^a^Effects were calculated and based on a linear mixed-effects model adjusted by manufacturers, occupations, sex, blood type, interval between two doses of vaccination, duration after vaccination, and body mass index. The log-transformed B lymphocyte count was the independent variable.

Abbreviations: CI: confidence interval; LSMD: least-square mean difference.

**Supplementary Table 12.** Kinetics of the level of IL-6^a^ according to age after vaccination with the Chinese inactive COVID-19 vaccine.

| Factors | Days post-vaccination | | | | | | | | | |
| --- | --- | --- | --- | --- | --- | --- | --- | --- | --- | --- |
|  | 1–14 | 15–30 | 31–60 | 61–90 | 91–120 | 121–150 | 151–180 | 181–240 | *F* | *p* |
| Average | 0.3 ± 0.4 | −0.4 ± 0.4 | 0.14 ± 0.4 | 0.7 ± 0.4 | -0.2 ± 0.3 | 1.3 ± 0.3 | 1.1 ± 0.4 | −0.61 ± 0.3 | 11.25 | <0.001 |
| 18–30 years | −0.48 ± 0.69 | −0.46 ± 0.54 | −0.67 ± 0.47 | −0.39 ± 0.39 | −0.64 ± 0.31 | 0.38 ± 0.37 | 0.56 ± 0.56 | −0.59 ± 0.39 | 1.857 | 0.078 |
| 31–50 years | 0.62 ± 0.54 | −0.15 ± 0.51 | 0.27 ± 0.62 | 0.52 ± 0.33 | −0.16 ± 0.44 | 1.88 ± 0.39 | 1.35 ± 0.59 | −0.72 ± 0.33 | 7.116 | <0.001 |
| >50 years | 0.59 ± 0.64 | −0.47 ± 0.61 | −0.68 ± 0.57 | 1.59 ± 0.37 | −0.13 ± 0.62 | 2.49 ± 0.55 | 1.39 ± 0.67 | −0.67 ± 0.60 | 7.232 | <0.001 |
| LSMD (95% CI) | - | - | - | 1.1 (0.3–1.9) | - | 2.1 (0.9–3.4) | - | - |  |  |
| *F* | 0.559 | 0.194 | 0.57 | 13.2 | 1.189 | 11.5 | 0.226 | 0.428 |  |  |
| *p* | 0.234 | 0.994 | 0.989 | 0.001^b^, 0.004^c^ | 0.411 | 0.001^b^ | 0.634 | 0.94 |  |  |

^a^Effects were calculated and based on a linear mixed-effects model adjusted by manufacturers, occupations, sex, blood type, interval between two doses of vaccination, duration after vaccination, and body mass index. The log-transformed level of IL-6 was the independent variable.

^b^Age: 18–30 years vs. > 50 years.

^c^Age: 31–50 years vs. 18–30 years.

Abbreviations: CI: confidence interval; IL-6: interleukin-6; LSMD: least-square mean difference.

**Supplementary Table 13.** Kinetics of the level of IL-6^a^ according to the manufacturer after vaccination with the inactive Chinese COVID-19 vaccine.

| Factors | Days post-vaccination | | | | | | | | | |
| --- | --- | --- | --- | --- | --- | --- | --- | --- | --- | --- |
|  | 1–14 | 15–30 | 31–60 | 61–90 | 91–120 | 121–150 | 151–180 | 181–240 | *F* | *p* |
| Average | 0.3 ± 0.4 | −0.4 ± 0.4 | 0.14 ± 0.4 | 0.7 ± 0.4 | −0.2 ± 0.3 | 1.3 ± 0.3 | 1.1 ± 0.4 | −0.61 ± 0.3 | 11.25 | <0.001 |
| Same manufacturer | 0.54 ± 0.41 | −0.16 ± 0.39 | −0.37 ± 0.39 | 0.71 ± 0.30 | 0.1 ± 0.3 | 1.76 ± 0.33 | 0.99± 0.45 | −0.38 ± 0.28 | 10.82 | <0.001 |
| Mixed manufacturers | −0.44 ± 0.89 | −0.42 ± 0.65 | 0.1 ± 0.61 | 0.33 ± 0.36 | −0.58 ± 0.38 | 0.72 ± 0.42 | 2.72 ± 0.89 | −1.41 ± 1.76 | 2.86 | 0.009 |
| LSMD (95% CI) | - | - | - | - | - | 0.97 (0.2–1.8) | 1.88 (0.1–3.7) |  |  |  |
| *t* | 1.051 | 0.365 | 0.557 | 1.113 | 1.766 | 2.483 | 1.819 | 0.588 |  |  |
| *p* | 0.294 | 0.715 | 0.578 | 0.266 | 0.078 | 0.013 | 0.044 | 0.557 |  |  |

^a^Effects were calculated and based on a linear mixed-effects model adjusted by sex, age, blood type, occupation, interval between two doses of vaccination, duration after vaccination, and body mass index. The log-transformed IL-6 level was the independent variable.

Abbreviations: CI: confidence interval; IL-6: interleukin-6; LSMD: least-square mean difference.

**Supplementary Table 14.** Kinetics of the level of IL-6^a^ according to occupation after vaccination with the Chinese inactive COVID-19 vaccine.

| Factors | Days post-vaccination | | | | | | | | | |
| --- | --- | --- | --- | --- | --- | --- | --- | --- | --- | --- |
|  | 1–14 | 15–30 | 31–60 | 61–90 | 91–120 | 121–150 | 151–180 | 181–240 | *F* | *p* |
| Average | 1.9 ± 0.2 | 1.5 ± 0.2 | 1.4 ± 0.2 | 1.4 ± 0.2 | 1.3 ± 0.3 | 1.3 ± 0.2 | 1.1 ± 0.2 | 1.0 ± 0.2 | 11.25 | <0.001 |
| Health staff | 2.3 ± 0.2 | 2.1 ± 0.2 | 1.9 ± 0.2 | 1.8 ± 0.2 | 1.7 ± 0.3 | 1.7 ± 0.2 | 1.5 ± 0.4 | 1.4 ± 0.2 | 10.62 | <0.001 |
| Workers | 1.8 ± 0.8 | 1.3 ± 0.5 | 0.8 ± 0.8 | 0.7 ± 0.5 | 0.7 ± 0.6 | 0.7 ± 0.3 | 0.6 ± 0.8 | 0.5 ± 0.6 | 1.13 | 0.371 |
| LSMD (95% CI) | 0.8 (0.6–1.2) | 0.8 (0.3–1.1) | 1.1 (0.6–1.9) | 1.1 (0.3–1.8) | 1.0 (0.3–1.7) | 1.0 (0.3–2.1) | 0.9 (0.2–5.5) | 0.9 (0.4–2.4) |  |  |
| *t* | 2.502 | 1.244 | 1.99 | 1.488 | 0.804 | 1.184 | 1.154 | 2.772 |  |  |
| *p* | 0.005 | 0.146 | 0.015 | 0.278 | 0.399 | 0.735 | 0.129 | 0.028 |  |  |

^a^Effects were calculated and based on a linear mixed-effects model adjusted by manufacturers, sex, age, blood type, interval between two doses of vaccination, duration after vaccination, and body mass index. The log-transformed level of IL-6 was the independent variable.

Abbreviations: CI: confidence interval; IL-6: interleukin-6; LSMD: least-square mean difference.

**Supplementary Table 15.** Kinetics of the level of IL-6^a^ according to interval between two doses after vaccination with the Chinese inactive COVID-19 vaccine.

| Factors | Days post-vaccination | | | | | | | | | |
| --- | --- | --- | --- | --- | --- | --- | --- | --- | --- | --- |
|  | 1–14 | 15–30 | 31–60 | 61–90 | 91–120 | 121–150 | 151–180 | 181–240 | *F* | *p* |
| Average | 1.9 ± 0.2 | 1.5 ± 0.2 | 1.4 ± 0.2 | 1.4 ± 0.2 | 1.3 ± 0.3 | 1.3 ± 0.2 | 1.1 ± 0.2 | 1.0 ± 0.2 | 11.25 | <0.001 |
| 21-40 days | 2.0 ± 0.2 | 1.9 ± 0.2 | 1.6 ± 0.2 | 1.6 ± 0.2 | 1.6 ± 0.2 | 1.4 ± 0.2 | 1.3 ± 0.3 | 1.2 ± 0.2 | 10.62 | <0.001 |
| >40 days | 1.9 ± 0.3 | 1.4 ± 1.2 | 1.2 ± 0.3 | 1.1 ± 0.3 | 0.9 ± 0.4 | 0.7 ± 0.5 | 0.7 ± 0.8 | 0.4 ± 0.4 | 1.13 | 0.371 |
| LSMD (95% CI) | 0.1 (0.1–0.8) | 0.5 (0.2–0.6) | 0.4 (0.2–0.7) | 0.5 (0.0–0.9) | 0.7 (0.2–0.9) | 0.7 (0.3–2.1) | 0.6 (0.3–2.5) | 0.8 (0.4–2.4) |  |  |
| *t* | 2.502 | 1.244 | 0.169 | 1.488 | 0.804 | 2.184 | 3.154 | 2.772 |  |  |
| *p* | 0.006 | 0.483 | 0.107 | 0.982 | 0.133 | 0.025 | 0.278 | 0.464 |  |  |

^a^Effects were calculated and based on a linear mixed-effects model adjusted by manufacturers, sex, age, blood type, occupation, duration after vaccination, and body mass index. The log-transformed level of IL-6 was the independent variable.

Abbreviations: CI: confidence interval; IL-6: interleukin-6; LSMD: least-square mean difference.

1. [↑](#footnote-ref-1)
